# Acidic pH reduces VEGF-mediated endothelial cell responses by downregulation of VEGFR-2; relevance for anti-angiogenic therapies

**DOI:** 10.18632/oncotarget.13323

**Published:** 2016-11-12

**Authors:** Seraina Faes, Emilie Uldry, Anne Planche, Tania Santoro, Catherine Pythoud, Nicolas Demartines, Olivier Dormond

**Affiliations:** ^1^ Department of Visceral Surgery, University Hospital of Lausanne, Switzerland

**Keywords:** acidity, VEGF, angiogenesis, sunitinib, endothelium

## Abstract

Anti-angiogenic treatments targeting the vascular endothelial growth factor or its receptors have shown clinical benefits. However, impact on long-term survival remains limited. Solid tumors display an acidic microenvironment that profoundly influences their biology. Consequences of acidity on endothelial cells and anti-angiogenic therapies remain poorly characterized and hence are the focus of this study. We found that exposing endothelial cells to acidic extracellular pH resulted in reduced cell proliferation and migration. Also, whereas VEGF increased endothelial cell proliferation and survival at pH 7.4, it had no effect at pH 6.4. Furthermore, in acidic conditions, stimulation of endothelial cells with VEGF did not result in activation of downstream signaling pathways such as AKT. At a molecular level, acidity significantly decreased the expression of VEGFR-2 by endothelial cells. Consequently, anti-angiogenic therapies that target VEGFR-2 such as sunitinib and sorafenib failed to block endothelial cell proliferation in acidic conditions. *In vivo*, neutralizing tumor acidity with sodium bicarbonate increased the percentage of endothelial cells expressing VEGFR-2 in tumor xenografts. Furthermore, combining sodium bicarbonate with sunitinib provided stronger anti-cancer activity than either treatment alone. Histological analysis showed that sunitinib had a stronger anti-angiogenic effect when combined with sodium bicarbonate. Overall, our results show that endothelial cells prosper independently of VEGF in acidic conditions partly as a consequence of decreased VEGFR-2 expression. They further suggest that strategies aiming to raise intratumoral pH can improve the efficacy of anti-VEGF treatments.

## INTRODUCTION

Targeting the formation of new blood vessels in tumors has shown clinical benefits in cancer patients [1, 2]. So far, most anti-angiogenic therapies have focused on the vascular endothelial growth factor (VEGF) and its receptors since they play a central role in angiogenesis [3]. Both antibodies targeting the soluble form of VEGF and small tyrosine kinase inhibitors of VEGF receptors have shown anti-tumor activity, yielding a significant increase in progression free survival in several types of cancer including advanced renal cell carcinoma [4, 5], advanced hepato-cellular carcinoma [6], and metastatic colorectal cancer [7]. The effect is however limited and tumors eventually escape the inhibition of VEGF signaling. In fact, tumors can use multiple angiogenic factors besides VEGF to promote tumor angiogenesis or switch to other modes of vascularization such as vascular mimicry [8]. Hence, resistances to anti-VEGF therapies have considerably limited their effectiveness. Identifying these resistance mechanisms will help design novel therapeutic approaches aiming to enhance efficacy of VEGF targeting therapies.

Tumor microenvironment is classically acid as a consequence of high rate of glucose metabolism and poor tumor perfusion [9]. Acidity offers tumor cells a growth advantage as, in contrast to other cell types present in the tumor microenvironment, cancer cells possess all the enzymatic machinery necessary to keep a physiological intracellular pH in acidic conditions [10, 11]. In addition, acidity favors tumor progression by increasing tumor cell mobility, invasion and metastasis [12, 13]. Furthermore, acidity participates in tumor immune escape by promoting T cell anergy [14]. Besides, acidic pH affects the response of cancer cells to conventional therapies. For instance, it reduces the efficacy of weak base chemotherapies by reducing their cellular uptake [15, 16]. Moreover, it renders cancer cells resistant to radiotherapy [17].

Whereas the effect of low pH values on cancer cells have been well characterized, little is known about the influence of acidity on endothelial cells and particularly on anti-VEGF therapies. In this study, we show that acidic extracellular pH decreases endothelial cell proliferation and abolishes VEGF-induced endothelial cell responses. In addition, acidity decreases the expression of VEGFR-2 by endothelial cells and consequently prevents the anti-angiogenic effect of sunitinib and sorafenib, two small tyrosine kinase inhibitors of VEGFR-2. *In vivo*, treating tumor bearing mice with sodium bicarbonate to raise the tumor microenvironmental pH increases the percentage of blood vessels expressing VEGFR-2 and potentiates the anti-cancer effects of sunitinib.

## RESULTS

### Acidity reduces endothelial cell (EC) proliferation and migration

**Figure 1:**
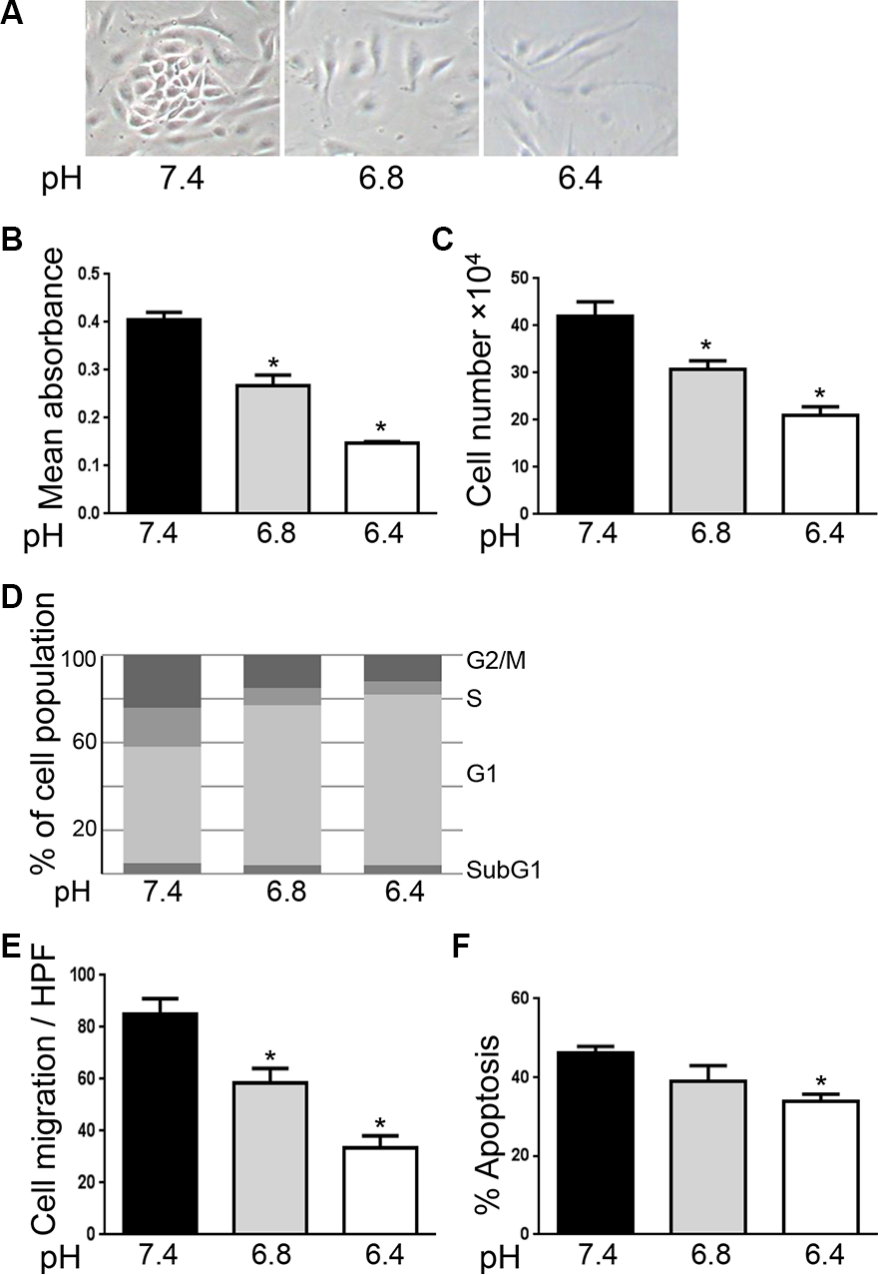
Acidity reduces endothelial cell proliferation and migration (**A**) Representative images of endothelial cells exposed to cultured medium buffered to pH 7.4, 6.8 or 6.4 for 48 hours. (**B**) MTS proliferation assay of EC cultured in medium buffered to the indicated pH for 48 hours. Results are expressed as mean absorbance at 490 nm of 10 independent experiments ± 1 SD. *p < 0.0001, Student’s *t-test*, compared to EC cultured at pH 7.4. (**C**) Endothelial cell count of EC cultured at the indicated pH for 48 hours. Dashed line represents the number of EC at the beginning of the experiments. Results are expressed as mean cell count ± 1 SD of 10 independent experiments. *p < 0.0001, Student’s *t-test*, compared to EC cultured at pH 7.4. (**D**) Cell cycle analysis using flow cytometry of endothelial cells treated as in panel a. One of three similar experiments is shown. (**E**) Migration assay of EC treated as in panel a. Results are expressed as mean cell count ± 1 SD per three fields at high power magnification (× 400). *p < 0.05, Student’s *t-test*, compared to EC cultured at pH 7.4. (**F**) Percentage of EC undergoing apoptosis following the withdrawal of serum and cultured at the indicated pH for 48 hours. Results are expressed as mean apoptosis percentage ± 1 SD of three independent experiments. *p < 0.05, Student’s *t-test*, compared to EC cultured at pH 7.4.

We first evaluated the consequences of exposing EC to extracellular acidity on cell functions that are relevant to angiogenesis including proliferation, migration and survival. EC exposed to extracellular acidity were either elongated or displayed a pancake like morphology compared to the cobblestone appearance of control EC (Figure 1A). Proliferation of EC was markedly reduced when EC were cultured in acidic conditions (Figure 1B). MTS proliferation assay showed a 64% and a 34% proliferation reduction when EC were cultured at pH 6.4 and 6.8 respectively, compared to EC cultured at pH 7.4 (*p < 0.0001*, *n* = 10). Similar results were obtained by cell counting (Figure 1C). Cell cycle analysis by flow cytometry showed that EC exposed to acidity displayed an increase in G1 phase cells associated with a decrease in S and G2/M phase cells. Of note, no changes in sub-G1 phase cells were observed (Figure 1D). Acidity also significantly decreased EC migration. As shown in Figure 1E, we found a 60% migration diminution when EC were cultured at pH 6.4 compared to 7.4 (*p < 0.05*, *n* = 3) and a 32% reduction at pH 6.8 compared to 7.4 (*p < 0.05*, *n* = 3). In addition, EC cultured in acidic conditions were more resistent to serum starvation induced apoptosis (Figure 1F). Serum withdrawal resulted in 46% of cells undergoing apoptosis after 48 hours when cultured at pH 7.4 versus 34% when cultured at pH 6.4 (*p < 0.05*, *n* = 3). Finally, acidity did not modify EC sprouting and tubulogenesis (data not shown). Taken together these results illustrate that extracellular acidity significantly impacts on EC functions relevant to angiogenesis.

Besides providing oxygen and nutrients to tissues, EC also actively participate in inflammation by recruiting leucocytes into sites of inflammation. The process of recruitment requires the production of chemokines and induction of ICAM-1 and VCAM-1 on EC which is mediated by factors that activate NF-κB, including TNF-α [18]. We thus next investigated whether acidity interferes with the response of EC to pro-inflammatory stimuli. To test this, EC were exposed to physiological pH or acidity and stimulated with TNF-α. Surface expression of ICAM-1 and VCAM-1 and intra-cellular expression of the chemokine MCP-1 were determined by flow cytometry. We found that VCAM-1 was not expressed by resting EC cultured either in physiological or acidic conditions. Expression levels of ICAM-1 and MCP-1 were similar in both conditions. TNF-α increased the expression of ICAM-1, VCAM-1 and MCP-1 similarly in EC exposed to acidity and in EC cultured at physiological pH (Supplementary Figure S1A–S1B).

### Acidity reduces VEGF-induced endothelial cell responses

**Figure 2:**
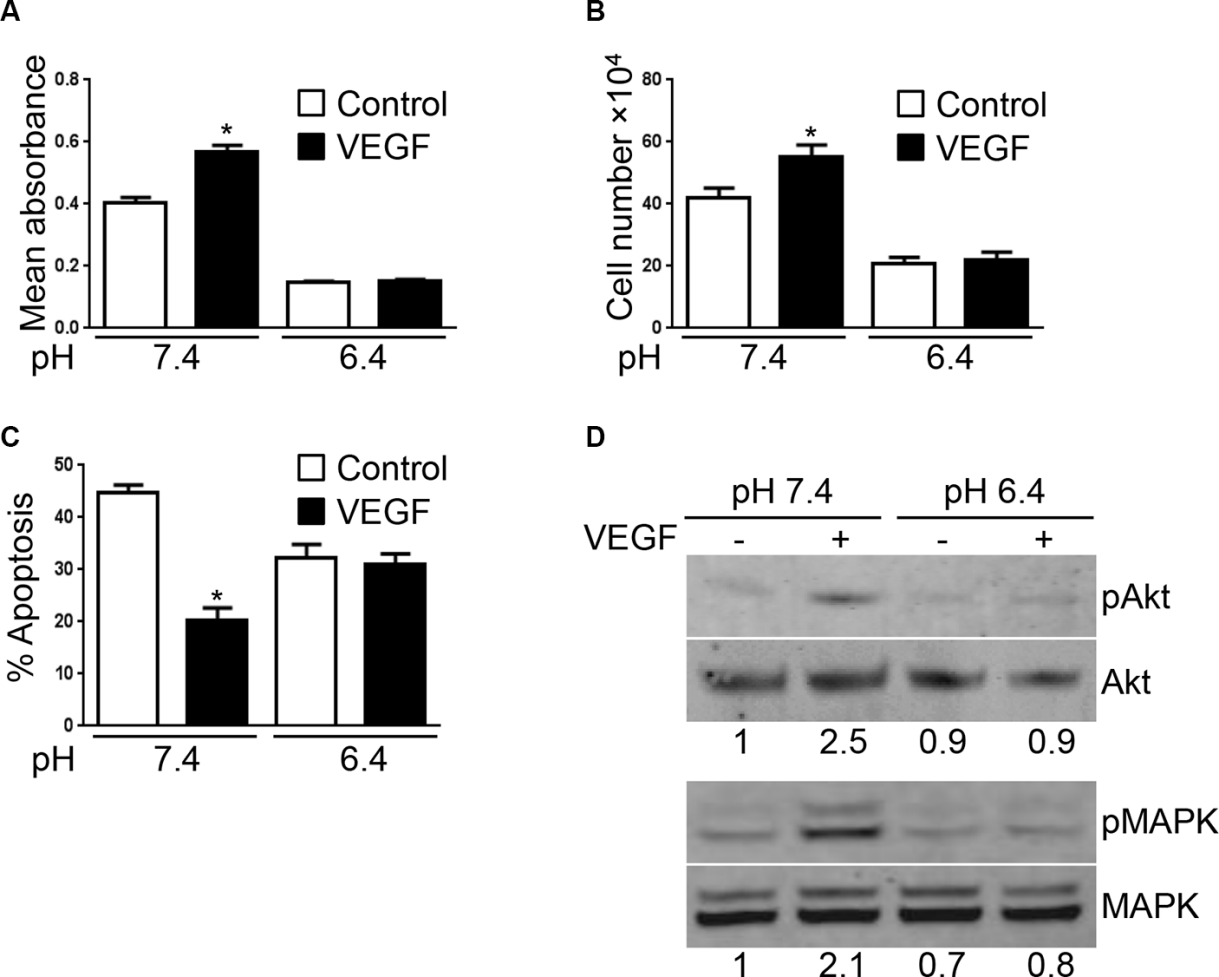
VEGF-mediated EC responses are inhibited by acidity (**A**) MTS proliferation assay of EC cultured at the indicated pH in presence or not of VEGF (10 ng/ml) for 48 hours. Results are expressed as mean absorbance at 490 nm of 10 independent experiments ± 1 SD. *p < 0.0001, Student’s *t-test*, compared to control EC cultured at pH 7.4. (**B**) Endothelial cell count of EC cultured at the indicated pH for 48 hours stimulated or not with VEGF (10 ng/ml). Results are expressed as mean cell count ± 1 SD of 10 independent experiments. *p < 0.0001, Student’s *t-test*, compared to control EC cultured at pH 7.4. (**C**) Percentage of EC undergoing apoptosis following the withdrawal of serum and cultured at the indicated pH for 48 hours ± VEGF stimulation (10 ng/ml). Results are expressed as mean apoptosis percentage ± 1 SD of three independent experiments. *p < 0.05, Student’s *t-test*, compared to control EC cultured at pH 7.4. (**D**) EC cultured at the indicated pH for 24 hours and subsequently stimulated or not with VEGF (10 ng/ml) for one hour. Cell lysates were prepared and analyzed by Western blot with the indicated antibodies. Densitometric values of the ratio of phosphorylated protein to total protein are listed below each blots.

VEGF acts as a major pro-angiogenic factor in part by inducing endothelial cell proliferation and survival [19]. Thus, we next wished to examine if acidity affects VEGF-induced endothelial cell responses. EC were exposed to acidic pH for 12 hours before stimulation with VEGF and cell proliferation was determined after 48 hours by MTS proliferation assay. As expected we found that VEGF increased EC proliferation by 32% (*p* < 0.0001) under physiological pH (Figure 2A). This effect was however lost when EC were cultured at pH 6.4. Similar results were obtained by cell counting (Figure 2B). Furthermore, VEGF protecting effect against serum starvation induced apoptosis was abolished when EC were cultured at pH 6.4. At pH 7.4, in serum starvation conditions for 48 hours, 45% of EC underwent apoptosis versus 20% in the presence of VEGF (*p < 0.05*). In contrast, in acidic conditions, 32% of EC were apoptotic compared to 31% in the presence of VEGF (Figure 2C).

Upon binding to its receptor VEGFR-2, VEGF triggers various signaling pathways. Among the different proteins activated by VEGFR-2-VEGF interaction, AKT and MAPK regulate EC proliferation [20–22]. We thus tested the ability of VEGF to activate AKT and MAPK in EC exposed to physiological or acidic pH. In Western blot, we found that VEGF increased AKT and MAPK phosphorylation at pH 7.4. This effect was however absent in EC cultured at pH 6.4 (Figure 2D).

Finally, to rule out that the lack of VEGF effects in acidic conditions was not due to VEGF inactivation by acidity, we set up the following experiment. EC were cultured at pH 7.4 or 6.4. After 24 hours of incubation, EC were collected and cultured for an additional 24 hours at pH 7.4 in the presence or not of VEGF. EC proliferation was then determined by MTS proliferation assay (Supplementary Figure S2A). We found that, as expected, VEGF increased the proliferation of EC that were cultured at pH 7.4. In contrast, VEGF had no effect on EC that were pre-exposed to pH 6.4 (Supplementary Figure S2B). Using the same experimental setting, we also investigated the protective effect of VEGF against serum starvation induced apoptosis. Similarly to what we found for EC proliferation, we observed that, whereas VEGF reduced the apoptosis rate of EC cultured at pH 7.4, it had no protective effect on EC that were pre-exposed to pH 6.4 (Supplementary Figure S2C).

### Acidity reduces VEGFR-2 expression by EC

**Figure 3:**
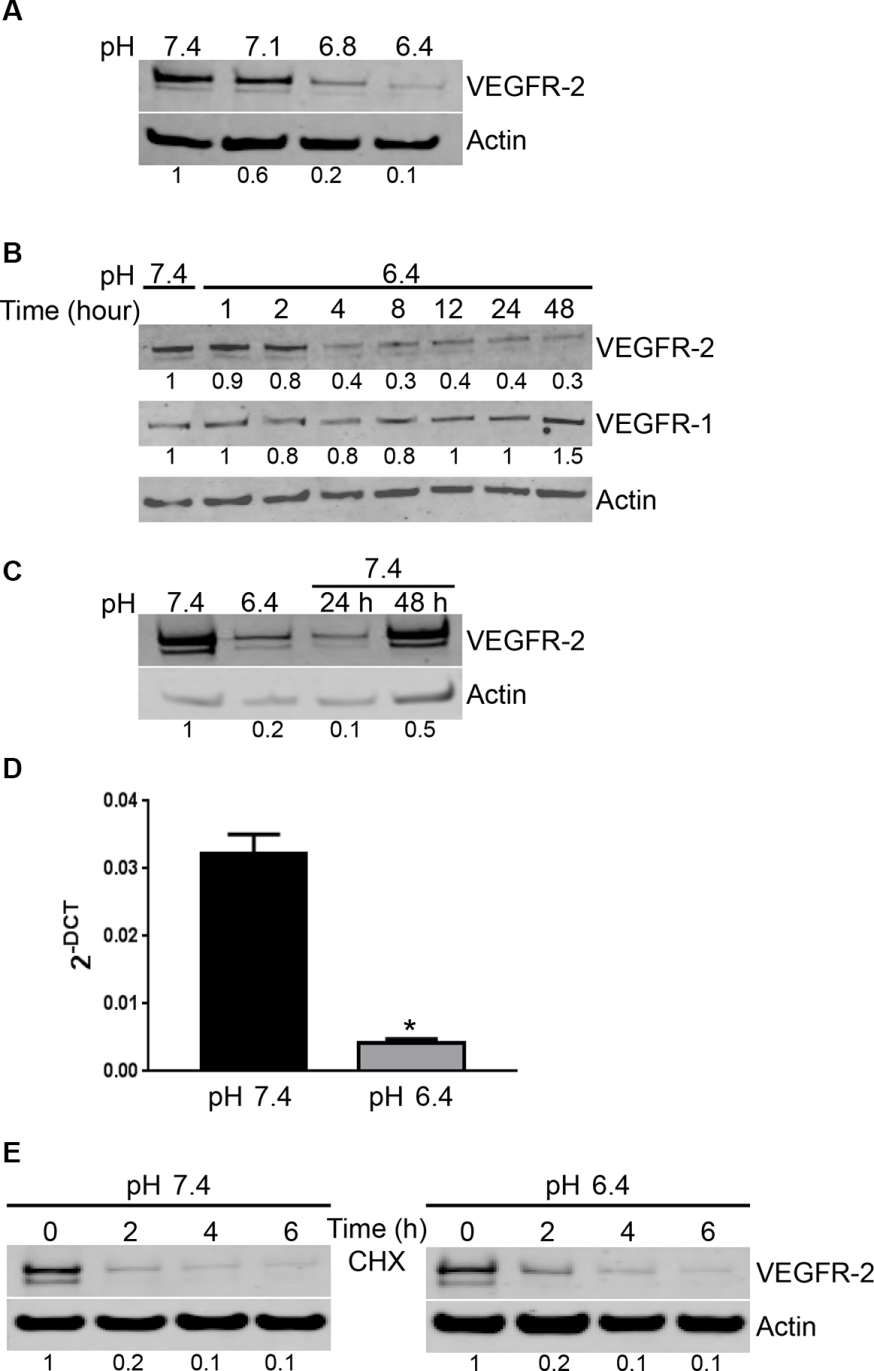
Acidity induces a reversible decrease of VEGFR-2 expression by EC (**A**) EC were cultured at the indicated pH for 24 hours. Cell lysates were analyzed by Western blot for VEGFR-2 and actin expression. Densitometric values of the ratio of VEGFR-2 to actin are listed below the blots. (**B**) EC were cultured at pH 6.4 for the indicated hours. Cell lysates were analyzed by Western blot for VEGFR-2, VEGFR-1 and actin expression. Densitometric values of the ratio of VEGFR-2 to actin and VGFR-1 to actin are listed below each blots. (**C**) EC were cultured for 24 hours at pH 6.4 followed by restoration at pH 7.4 for 24 and 48 hours. Cell lysates were analyzed by Western blot for VEGFR-2 and actin expression. Densitometric values of the ratio of VEGFR-2 to actin are listed below the blots. (**D**) EC were exposed to pH 6.4 or physiological pH for 6 hours. Total mRNA was extracted and tested for VEGFR-2 levels and cyclophilin as a control by real-time PCR. Bar charts represent mean, error bars represent SD. *p < 0.05, Student’s *t-test*, compared to EC cultured at pH 7.4. (**E**) EC were cultured at pH 7.4 or 6.4 for the indicated times in the presence of cycloheximide (CHX; 10 μM). Cell lysates were analyzed by Western blot for VEGFR-2 and actin expression. Densitometric values of the ratio of VEGFR-2 to actin are listed below the blots.

To gain insight into the mechanisms responsible for the loss of effect of VEGF in acidic conditions, we determined the level of expression of VEGF receptors by EC. VEGF possesses three major receptors VEGFR-1, VEGFR-2 and VEGFR-3, with VEGFR-3 acting mostly on lymphatic endothelial cells [23]. Using Western blot, we found that EC cultured in acidic conditions significantly reduced the expression of VEGFR-2. The reduction was apparent at pH 6.8 and maximal at pH 6.4 (Figure 3A). Decreased expression of VEGFR-2 took already place after 4 hours of EC exposure to pH 6.4 (Figure 3B). In contrast, acidity did not alter the expression of VEGFR-1 (Figure 3B). The loss of VEGFR-2 expression was reversible; however, EC required 48 hours at pH 7.4 to reincrease their levels of VEGFR-2 (Figure 3C). To clarify the mechanism implicated in the reduction of VEGFR-2 expression induced by acidity, we determined VEGFR-2 mRNA level using real-time PCR. We found that VEGFR-2 mRNA was significantly reduced in EC exposed to pH 6.4 compared to pH 7.4 (Figure 3D). We further investigated the effects of acidic pH on VEGFR-2 degradation by treating EC with cycloheximide to block protein synthesis. Low pH had no significant effects on VEGFR-2 degradation, as in both, acidic and physiological pH, VEGFR-2 was mostly degraded after two hours of treatment (Figure 3E).

We further investigated whether the effect of acidity was specific for VEGFR-2 or whether it also affected the expression of other molecules implicated in angiogenesis, including FGFR-1, Tie-2, CD31 or avβ3 integrin. Using flow cytometry, we found that acidity did not change the expression of CD31 and avβ3 integrin (Supplementary Figure S3A). Furthermore, we observed that acidity did not alter the expression levels of FGFR-1 and Tie-2 as evidenced by Western blot (Supplementary Figure S3B). All together these data suggest that acidity specifically down-regulates the expression of VEGFR-2 in EC.

### Acidity reduces the anti-proliferative efficacy of anti-VEGF therapies *in vitro*

**Figure 4:**
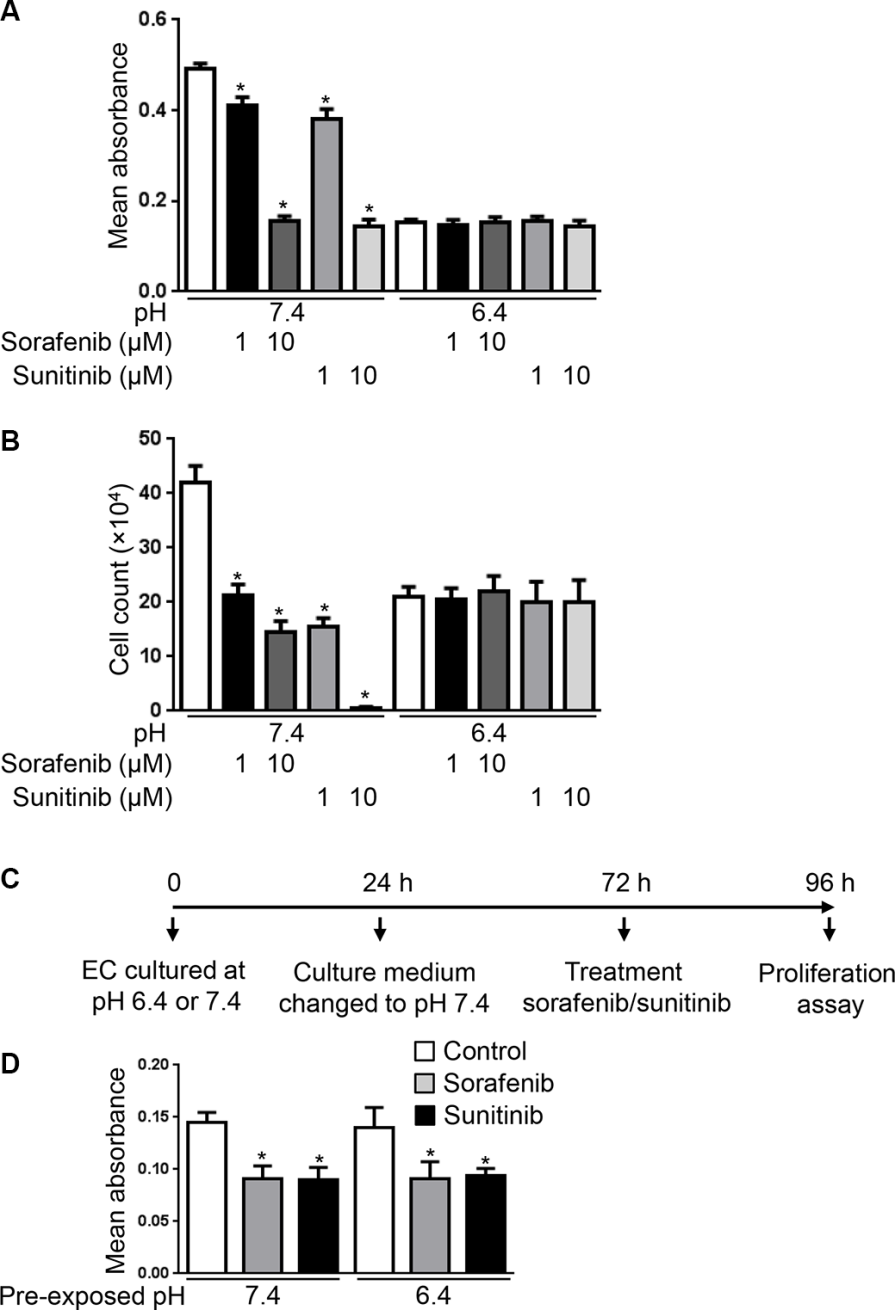
Acidity decreases the anti-proliferative efficacy of sorafenib and sunitinib (**A**) MTS proliferation assay of EC cultured at the indicated pH in presence or not of sorafenib (1 or 10 μM) or sunitinib (1 or 10 μM) for 48 hours. Results are expressed as mean absorbance at 490 nm of 10 independent experiments ± 1 SD. *p < 0.0001, Student’s *t-test*, compared to control EC cultured at pH 7.4. (**B**) Endothelial cell count of EC cultured at the indicated pH for 48 hours in presence or not of sorafenib (1 or 10 μM) or sunitinib (1 or 10 μM). Results are expressed as mean cell count ± 1 SD of 10 independent experiments. *p < 0.0001, Student’s *t-test*, compared to control EC cultured at pH 7.4. (**C**) Experimental set-up of the experiment presented in panel d. EC were exposed to pH 7.4 or 6.4 for 24 hours followed by restoration of pH 7.4 for 48 hours. EC were subsequently collected and plated for a 24 hour proliferation assay at pH 7.4 in presence or not of sunitinib (1 μM) or sorafenib (1 μM). (**D**) MTS proliferation assay of EC processed as described in panel c. Results are expressed as mean absorbance at 490 nm of triplicates ± 1 SD. *p < 0.05, Student’s *t-test*, compared to control EC cultured at pH 7.4 or 6.4.

We next hypothesized that since VEGFR-2 expression is reduced in EC exposed to pH 6.4, therapies that target VEGFR-2 would lose their efficacy in acidic conditions. To test this, EC were cultured at pH 7.4 or 6.4 and treated with sorafenib or sunitinib, two small molecule receptor tyrosine kinase inhibitors targeting VEGFR-2 [24, 25]. MTS proliferation assay performed after 48 hours of treatment revealed that sorafenib and sunitinib significantly reduced EC proliferation at pH 7.4 (Figure 4A). We found 17% and 68% proliferation reduction by sorafenib and 23% and 71% proliferation reduction by sunitinib respectively at doses of 1 and 10 μM compared to untreated EC (*p < 0.0001*). In contrast, no significant changes of proliferation were found when EC were exposed to pH 6.4 (Figure 4A). Similar results were obtained by cell counting (Figure 4B).

To exclude that the loss of antiproliferative effects of sorafenib and sunitinib in acidic conditions being due to their inactivation by acidity, we prexposed EC for 24 hours to culture medium buffered to pH 6.4 and subsequently treated EC and performed the proliferation assay at pH 7.4 (Supplementary Figure S4A). According to our Western blot analysis, VEGFR-2 expression is reduced in acidic conditions after 24 hours and its expression following restoration to physiological pH needs more than 24 hours (Figure 3C). In this experimental setting, we found that sorafenib and sunitinib did not reduce EC proliferation when EC were pre-exposed to pH 6.4, hence ruling out that the loss of activity in acidic conditions did result from their inactivation by acidity (Supplementary Figure S4B).

We further investigated whether the reduced anti-proliferative efficacy of sorafenib and sunitinib on EC cultured in acidic conditions can be reversed by re-exposing EC to physiological pH. To test this, EC were cultured at pH 6.4 for 24 hours followed by 48 hours culture at pH 7.4. At that time EC were collected and plated for a proliferation assay performed in the presence or absence of sorafenib or sunitinib (Figure 4C). We chose 48 hours restoration at physiological pH as it is the time required for EC to re-express VEGFR-2 (Figure 3C). We observed that the proliferation rate of EC that were exposed to acidity was restored following exposure to pH 7.4. We further found that sorafenib and sunitinib significantly reduced EC proliferation following restoration of pH 7.4 for 48 hours (Figure 4D). Altogether these data suggest that acidity reduces the anti-proliferative efficacy of sorafenib and sunitinib. They further suggest that maintaining a physiological pH is necessary to maintain their efficacy.

### Sodium bicarbonate potentiates the anti-cancer efficacy of sunitinib *in vivo*

**Figure 5:**
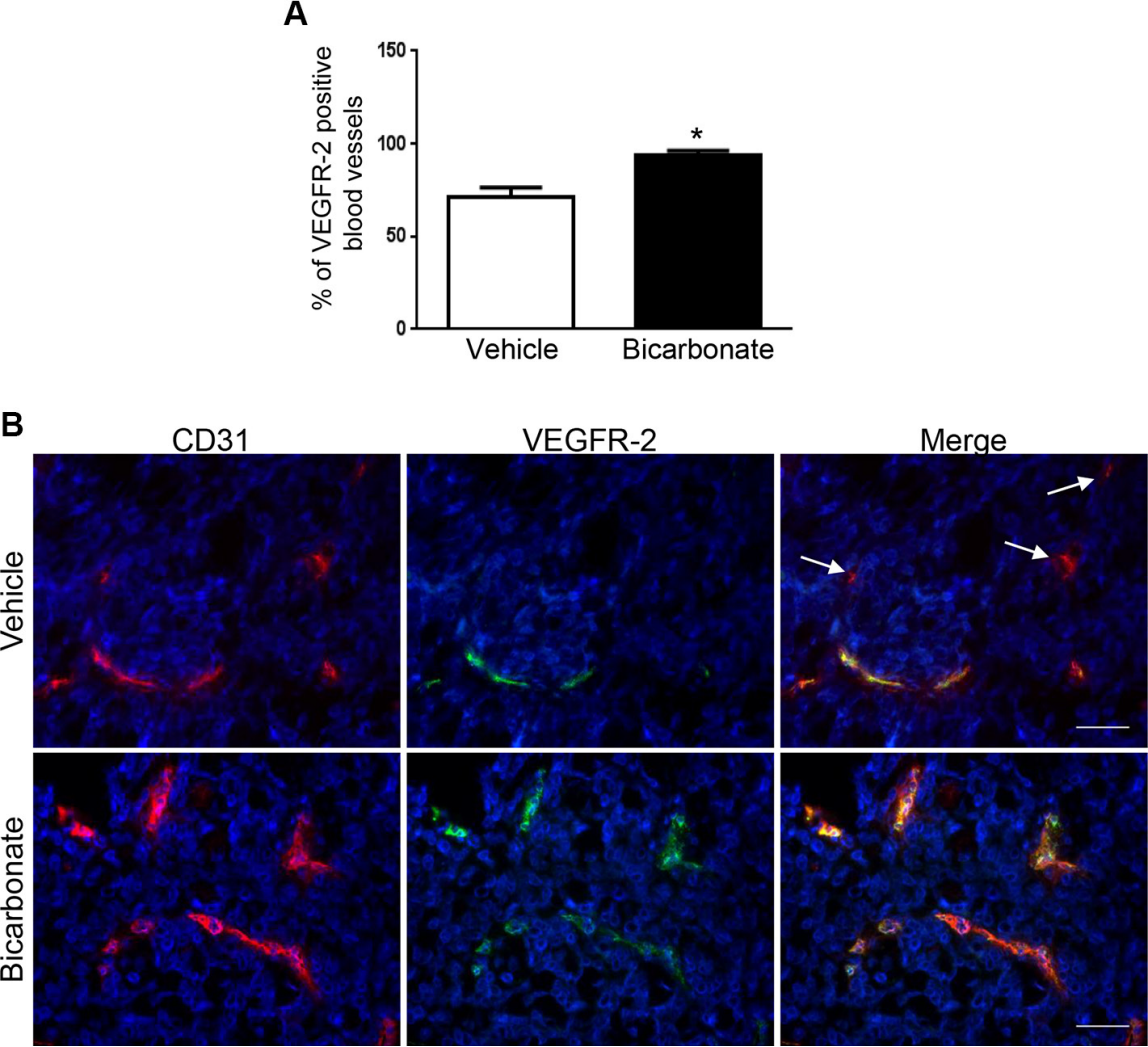
Sodium bicarbonate increases the percentage of VEGFR-2 positive blood vessels in HT29 tumor xenografts (**A**) Mean percentage ± 1 SD of VEGFR-2 positive blood vessels in HT29 tumor xenografts grown in nude mice left untreated (vehicle) or receiving sodium bicarbonate in the drinking water (bicarbonate; 200 mmol/L). (**B**) Dual immunofluorescent staining for CD31 (red) and VEGFR-2 (green) in HT29 tumor xenografts harvested from nude mice that were untreated (vehicle) or received sodium bicarbonate (bicarbonate). Arrows: example of CD31 positive, VEGFR-2 negative blood vessels. Scale bars, 50 μM.

Our *in vitro* observations suggest that acidity reduces the sensitivity of EC to sunitinib and sorafenib by reducing the expression of VEGFR-2. To next investigate the relevance of our findings *in vivo*, we hypothesized that increasing the intra-tumoral pH would increase VEGFR-2 on blood vessels and hence potentiate the anti-angiogenic efficacy of anti-VEGFR-2 therapies. Previous reports demonstrated that the acidic extracellular pH of tumors can be safely increased by sodium bicarbonate in tumor xenografts [26]. We thus grew HT29 colon cancer cell tumor xenografts in nude mice and treated them with sodium bicarbonate. Following tumor harvesting dual immunofluorescence staining for CD31 and VEGFR-2 was performed to determine VEGFR-2 expression on CD31 blood vessels. We found that 71.6% of CD31 blood vessels expressed VEGFR-2 in untreated mice (Figure 5A–5B). The percentage rose to 93.9% when mice received sodium bicarbonate (*p < 0.05* compared to untreated mice).

**Figure 6:**
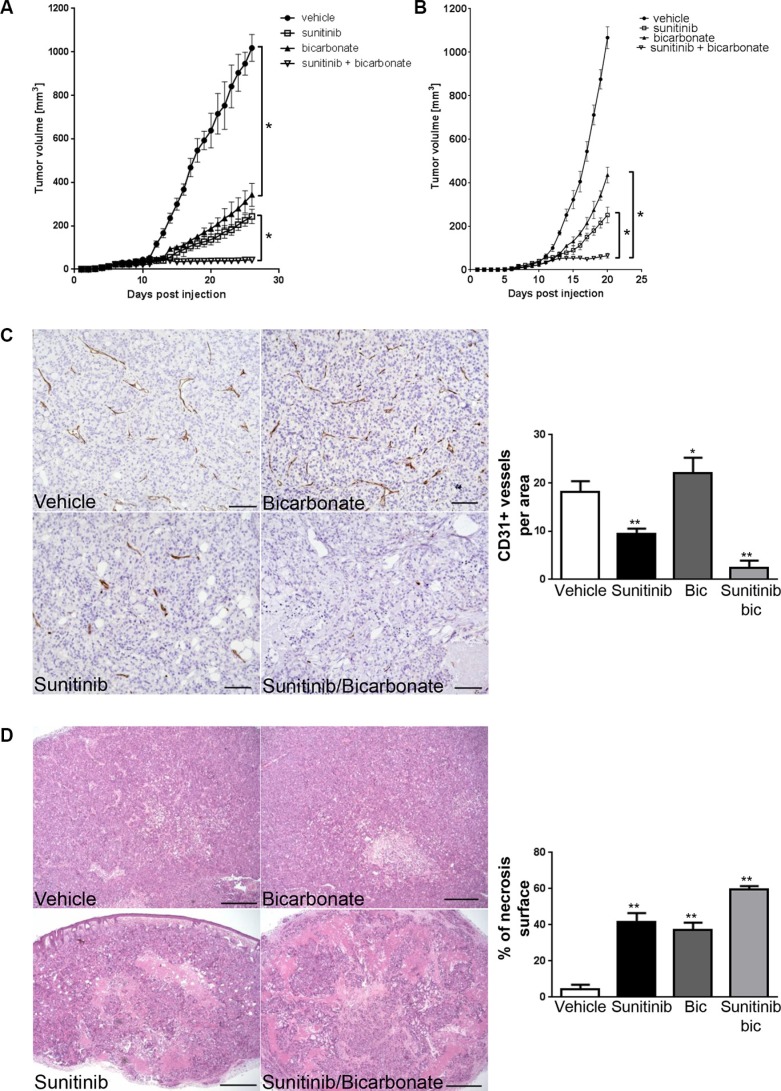
Sodium bicarbonate potentiates the anti-angiogenic efficacy of sunitinib (**A**) HT29 xenograft growth curves for treatments with vehicle, sunitinib (40 mg/kg p.o.), sodium bicarbonate (200 mmol/L in the drinking water) or a combination of both. *p < 0.0001, *n* = 5/group, Two-way ANOVA. (**B**) MC-38 allograft growth curves for treatments with vehicle, sunitinib (40 mg/kg p.o.), sodium bicarbonate (200 mmol/L in the drinking water) or a combination of both. * *p < 0.0001*, *n* = 5/group, Two-way ANOVA. (**C**) Tumor vasculature in HT29 xenografts was analyzed by counting CD31 positive vessels in 10 representative sections of 500 × 500 μm for three different tumors of each treatment group. Scale bars, 100 μm. Bar charts represent mean, error bars represent SD. **p < 0.0001, *p < 0.05, One-way ANOVA. (**D**) Tumor necrosis in HT29 xenografts (light pink stained surface in H&E) was evaluated in each treatment group in 10 representative sections of 3368 × 2668μm for three different tumors. Scale bars, 500 μm. Bar charts represent mean, error bars represent SD. **p < 0.0001, One-way ANOVA.

We next determined whether sodium bicarbonate could increase the anti-angiogenic and anti-tumor efficacy of sunitinib. To support this, we hypothesized that since the majority of blood vessels in tumor xenografts exposed to sodium bicarbonate are VEGFR-2 positive, blood vessels would be more sensitive to anti-VEGFR-2 therapy. To test this, mice bearing HT29 tumor xenografts were randomized into four treatment groups; vehicle, sodium bicarbonate, sunitinib, sodium bicarbonate and sunitinib. We found that tumor xenografts grew significantly slower in the sodium bicarbonate and sunitinib treatment groups. Combining sodium bicarbonate with sunitinib resulted in a stronger anti-cancer activity than either treatment alone (Figure 6A). Similar results were obtained in MC-38 tumor allografts (Figure 6B). Histological analysis revealed that combining sodium bicarbonate with sunitinib significantly decreased the number of blood vessels (87%) compared to sunitinib alone (48%) (Figure 6C). This was associated with increased necrosis (13.4 fold increase compared to vehicle, 1.6 fold increase compared to bicarbonate and 1.4 fold increase compared to sunitinib) (Figure 6D).

## DISCUSSION

Anti-angiogenic therapies are one of the most prescribed treatments in oncology. Indeed, up to ten drugs that target VEGF or its receptors have now been approved in cancer therapy [27]. However, to date, the anti-cancer efficacy of these compounds is limited and high effort is needed to improve their efficacy. In the present work, we have shown that acidity dampens the efficacy of anti-VEGF treatments. *In vitro*, acidic pH considerably reduced VEGF-mediated endothelial cell responses, suggesting that in acidic conditions endothelial cells prosper independently of VEGF. Accordingly, we found that low pH values significantly reduced the expression of VEGFR-2 by EC. *In vivo*, buffering tumor acidity with sodium bicarbonate increased the number of CD31/VEGFR-2 positive blood vessels in tumor xenograft and potentiated the anti-cancer efficacy of sunitinib. Hence, sodium bicarbonate might improve the efficacy of sunitinib by increasing the number of blood vessels that express VEGFR-2.

Tumor acidity profoundly influences the biology of tumors [10]. It affects cancer cells by increasing their motility and invasiveness and reduces the response of cancer cells to radiotherapy and weak base chemotherapies. Furthermore, acidity also modulates the tumor microenvironment by favoring T-cell anergy and hence promoting tumor immune escape [14]. Accordingly, the anti-tumor efficacy of immunotherapies is increased following the neutralization of tumor acidity [28]. Our study further highlights acidity as a factor that influences tumor angiogenesis. Indeed, we observed that acidity decreased endothelial cell proliferation and migration suggesting that acidity slows the process of angiogenesis. Accordingly we found that sodium bicarbonate significantly increased the number of blood vessels in tumor xenografts (Figure 6B). Consistent with our observations, it was reported in the rat aortic ring model that acidity induced a marked delay in microvascular growth [29]. Also, acidity delayed migration of endothelial cells in irradiated wounds [30].

EC show heterogeneity in structure and function and accordingly protein expression differs significantly [31]. For instance, the expression of VEGFR-2 or Tie-2 by tumor endothelial cells varies in tumors [32, 33]. Similarly, we found that VEGFR-2 was not expressed by every tumor blood vessel (Figure 5A). The therapeutic consequence of such heterogeneity remains however poorly characterized. Whilst it is known that tumors displaying higher degree of Tie-2 negative blood vessels respond less to anti-Tie2 therapy [32], little is known about the responsiveness of VEGFR-2 negative blood vessels to anti-VEGF treatment. Here, we find that increased numbers of VEGFR-2 expressing tumor blood vessels were associated with increased efficacy of sunitinib suggesting that acidity lessens the efficacy of anti-VEGF therapies by reducing VEGFR-2 expression. Of note, several resistances to anti-angiogenic drugs have been identified including escape via different modes of vascularization and secretion of multiple pro-angiogenic factors [34]. Since acidity profoundly affects the phenotype of cancer cells and cells present in the tumor microenvironment, several other mechanisms, not limited to an increased expression of VEGFR-2 by tumor EC, are expected to participate in the process of resistance to anti-VEGF therapies.

Recent studies have shown that sodium bicarbonate displays anti-cancer activity in different mouse models [35]. Sodium bicarbonate reduced the number and size of metastases in a breast tumor xenograft model [26]. In addition, it also reduced the formation of liver metastasis following cancer cell injection into the tail vein. Contrasting results exist on the effect of sodium bicarbonate on the growth of primary tumor in mice models. In human tumor xenografts, sodium bicarbonate reduced the growth of HCT-116 colon tumors but had no effect on the growth of MDA-MB231 breast tumors [26, 36]. Similarly, in immune competent C57BL/6 mice, bicarbonate therapy did not affect the growth rate of B16 melanoma tumors but significantly reduced the growth of Yumm1.1 melanoma [28]. Finally, in a transgenic model of prostate cancer, bicarbonate prevented the development of tumors [37]. In our study, we observed that sodium bicarbonate significantly reduced the growth rate of HT29 tumor xenografts and MC-38 tumor allografts. Hence, future studies are needed to fully characterize the anti-cancer efficacy of sodium bicarbonate and identify the molecular mechanisms responsible for these effects.

We found that sodium bicarbonate treatment increased VEGFR-2 expression and mean vessel density but reduced tumor growth. This was also associated with increased tumor necrosis. Such paradigm was previously reported in other experimental settings such as following DLL-4-Notch blockade and was due to immature, non functional blood vessels [38, 39]. In addition, other molecular mechanisms could contribute to the anti-cancer effects of sodium bicarbonate. It is well established that acidity impairs the function of other cell types present in the tumor microenvironment including inflammatory/immune cells. For instance, acidity increases the response of tumor promoting macrophages [40]. Also acidity induces anergy of tumor infiltrating T cells and reduces the activity of NK cells [14, 41]. Therefore, sodium bicarbonate could favor anti-tumor responses of immune cells by at least two mechanisms. Firstly, the augmented mean vascular density induced by sodium bicarbonate could increase the recruitment of these cells into the tumor and secondly buffering tumor acidity could increase their anti-tumor activity. Clearly additional studies are needed to fully characterize the mechanisms by which sodium bicarbonate reduces tumor growth.

The use of sodium bicarbonate in human patients might be associated with toxicity and needs to be evaluated. However, other means exist to target tumor acidity. For instance, emerging evidence have demonstrated the contribution of the carbonic anhydrase IX (CAIX) in creating an acidic tumor microenvironment [42]. Interestingly, it was shown that targeting CAIX enhances the efficacy of bevacizumab, a VEGF targeting antibody, further underlining a possible role for tumor acidity in decreasing the effects of anti-angiogenic drugs [43]. Several specific CAIX inhibitors have now been developed and are entering phase I clinical trials and thus warrant to be tested in combination with anti-angiogenic therapies [44]. Beside CAIX, many other proteins participate in the regulation of pH in tumors including V-ATPase, monocarboxylate transporters, Na^+^/H^+^ exchangers which could also be easily targeted [10, 11, 45].

In conclusion, our results show that acidity reduces VEGF-mediated endothelial cell responses *in vitro* and accordingly diminishes the anti-angiogenic efficacy of anti-VEGF therapies. They further provide a rationale to associate anti-angiogenic treatments with therapies aiming to increase intra-tumoral pH in clinical trials.

## MATERIALS AND METHODS

### Antibodies and reagents

Sunitinib and sorafenib were from LC Laboratories (Woburn, MA, USA). Sodium bicarbonate and HEPES (4-(2-hydroxyethyl)-1-piperazineethanesulfonic acid) were from Sigma-Aldrich. Recombinant human VEGF was from Peprotech (#100-20-10). Cycloheximide was purchased from Santa Cruz biotechnology (#SC-3508). For Western blot analysis, the following primary antibodies and concentrations were used: anti-phospho-Akt antibody (1:500) (#4060; Cell Signaling Technology), anti-Akt antibody (1:1000) (#2920; Cell Signaling Technology), anti-VEGFR-2 antibody (1:1000) (#2479; Cell Signaling Technology), anti-VEGFR-1 (1:500) (#sc-316; Santa Cruz biotechnology), anti-β-actin antibody (1:5000) (#A2228; Sigma Aldrich), anti-Tie-2 antibody (1:500) (#MAB313; R&D systems) and anti-FGFR-1 antibody (1:1000) (#sc-121; Santa Cruz Biotechnology). Immunohistochemical staining were performed with anti-CD31 antibody (#Rb-10333-PO; Thermo Scientific). For immunofluorescence anti-CD31 (1:50) (# 553370; BD Pharmigen) and anti-VEGFR-2 antibody (1:50) (#2479; Cell Signaling Technology) were used. For flow cytometry, the following antibodies and dilutions were used: anti-CD31 (1:100) (#17-0319; eBioscience), anti-avb3 integrin (1:100) (MAB1976; Millipore), anti-VCAM-1(1:100) (#12-1069; eBioscience), anti-ICAM-1 (1:100) (#12-0549; eBioscience) and anti-MCP-1 (1:100) (#12-7099; eBioscience).

### Cell culture

Human umbilical vein endothelial cells (HUVEC) were purchased from Lonza and cultured in EBM complete medium. HUVEC were used for the experiments between passages 2 and 5. HT29 human colon cancer cells were obtained from ATCC and murine colon adenocarcinoma cell line MC-38 was kindly provided by Dr. Jeffrey Schlom (National Cancer Institute, NIH) [46]. Both cell lines were cultured in Dulbecco’s Modified Eagle’s Medium - high glucose (DMEM) (Sigma-Aldrich, Buchs, Switzerland) supplemented with 10% FBS and 1% streptomycin/penicillin.

### Proliferation assay

EC were plated on 96 well plates (Costar) at 10’000 cells per well and cultured in EBM complete medium. Twelve hours later, medium was removed and replaced by EBM complete medium buffered to pH 7.4, 6.8 or 6.4. Cellular proliferation was monitored after 48 hours with CellTiter 96^®^ AQ_ueous_One Solution (Promega Corporation) colorimetric assay by following the manufacturer’s instructions. Results are expressed as mean absorbance at 490 nm of 10 independent experiments ± 1 SD.

### Cell cycle analysis

EC were cultured in medium buffered to pH 7.4, 6.8 or 6.4 for 48 hours. Attached and floating EC were then collected and fixed in 95% ethanol overnight. Cells were washed twice in phosphate buffered saline (PBS) and subsequently incubated in PBS containing propidium iodide (50 μg/ml) and RNase A (100 μg/ml). Cell cycle was analyzed by fluorescence-activated cell sorter using Cellquest software (BD Biosciences). For apoptosis assay, EC were cultured in serum free medium buffered to pH 7.4, 6.8 or 6.4 for 48 hours and subsequently analyzed as above.

### Cell count

One hundred thousand EC were plated in six well plates coated with gelatin 0.5%. After attachement, medium was replaced with cell medium buffered at pH 7.4, 6.8 or 6.4 for 48 hours. Subsequently, adherent cells were collected and trypan-blue negative cells were counted using a Neubauer hemocytometer. Results are expressed as mean cell count ± 1 SD of 10 independent experiments.

### Migration assay

Migration assays were performed as previously described [47]. Briefly, the lower surface of an 8 μm pores Transwell filter was coated with fibronectin (10 mg/ml) for two hours and subsequently blocked with 1% bovine serum albumin for one additional hour. Endothelial cells were exposed to the indicated pH for 48 hours, collected and added to the upper chamber of the transwell in serum free medium (4 × 10^4^ endothelial cells per transwell). After 3 hours filters were fixed in 2% paraformaldehyde and stained in 0.5% crystal violet. Migrated cells were counted on the lower surface of the filter by light microscopy in three high-power fields. Results are expressed as mean cell count ± 1 SD per three fields at high power magnification (× 400).

### Flow cytometry

EC were cultured for 24 hours at pH 7.4 or 6.4. For some experiments, EC were subsequently stimulated or not with TNF-α (10ng/ml) for twelve hours. EC were collected, rinsed and incubated in PBS with APC-conjugated antibody to CD31 (#17-0319; eBioscience), FITC-conjugated antibody to avb3 integrin (MAB1976; Millipore), phycoerythrin-conjugated antibody to VCAM-1 (#12-1069; eBioscience) or ICAM-1 (#12-0549; eBioscience) or with the labeled matched IgG isotype as control for 45 minutes at 4°C. Stained cells were analyzed in a FACSCalibur using CellQuest software (Becton Dickinson). To measure intracellular MCP-1 production, GolgiStop (#554715; BD Bioscience) was added to the cell culture medium 3 hours before analysis. Cells were fixed/permeabilized in Cytofix/Cytoperm solution (#554715; BD Bioscience) following the manufacturer’s instructions. Fixed cells were incubated with phycoerythrin-conjugated antibody to MCP-1 (#12-7099; eBioscience) or phycoerythrin-labeled matched IgG isotype as control for 45 minutes at 4°C and analyzed as above.

### Western blot analysis

EC were plated in 6 well plates at 200’000 cells per well and cultured in EBM medium adjusted to different pH using HEPES. For some experiments, EC were cultured at the indicated pH for 24 hours followed by a one hour stimulation with VEGF (10 ng/ml). Cells were lysed in RIPA buffer. Protein concentrations were measured using BCA Assay (Pierce, Rockford, IL, USA). Equal amounts of protein (20 μg) were separated on 4–12% polyacrylamide gel and subsequently transferred to a polyvinylidene difluoride membrane (Millipore, Schaffhausen, Switzerland). Membranes were blocked with Odyssey blocking buffer (LI-COR Biosciences, Lincoln, NE, USA) and immunoblotted with primary antibodies followed by infrared secondary antibodies. Bands from immunoreactive proteins were visualized by an Odyssey infrared imaging system (LI-COR Biosciences). Densitometric analysis was performed using ImageJ software. Density values of phosphorylated proteins were normalized to total protein for each sample. In some experiment, density values of protein were normalized to actin. Unstimulated cells were given a value of 1.0, and ratios in all other samples were normalized to this value. Densitometric values are listed below each blot.

### Real-time PCR

RNA extraction was performed using RNeasy Mini Kit from Qiagen by following the manufacturer’s instructions. We used 500 ng of RNA for reverse transcription with SuperScript II Reverse Transcriptase from ThermoFisher Scientific. The resulting cDNA was used for qRT-PCR (Rotor-Gene Q from Qiagen). qRT-PCR were set up in triplicates with KAPA SYBR FAST qPCR Kit Master Mix Universal KK4602 from Kapa Biosystems. The relative expression levels of the target gene mRNAs were calculated by the comparative C_T_ method (relative expression = 2^−ΔCT^) using cyclophilin as an internal control. Primer sequences were: human VEGFR2 forward ATC CCT GTG GAT CTG AAA CG, human VEGFR2 reverse CCA AGA ACT CCA TGC CCT TA, human VEGFA forward CCT CCG AAA CCA TGA ACT TT, human VEGFA reverse ATG ATT CTG CCC TCC TCC TT, human cyclophilin forward ACC GTG TTC TTC GAC ATT GC, human cyclophilin reverse TTA TGG CGT GTG AAG TCA CC.

### Immunohistochemistry

Xenografts were fixed in 4% formaline overnight, dehydrated with ethanol and paraffin-embedded. Sections of 3μm were obtained using MICROM HM 355S microtome (Thermo Scientific, Ecublens, Switzerland), and tissue sections were mounted on Superfrost Plus slides (Thermo Scientific, Ecublens, Switzerland). Slides were then deparaffinized and rehydrated with xylol and alcohol. After antigen retrieval (citrate pH 6.0 or TRIS/EDTA pH 9.0), sections were immunostained using anti-CD31 primary antibody for 60 minutes and subsequently incubated with Dako EnVision HRP secondary antibody (Dako, Baar, Switzerland) for 30 minutes. In parallel, staining with haematoxylin and eosin were performed. One section from each xenograft tumor and three tumors for each condition were analyzed for each staining. Carl Zeiss Axioscope, AxioCam MRc and AxioVision 40V 4.6.3.0 software (Carl Zeiss Vision Swiss AG, Feldbach, Switzerland) were used for image acquisition and processing. Histology analysis was performed by two researchers blinded to groupings. Blood vessel count was determined in 10 representative sections of 500 × 500 μm for three different tumors of each treatment group. Percentage of tumor necrosis (light pink stained surface in H&E) were measured quantitatively using ImageJ 1.46r Threshold Colour Plugin by analyzing 10 representative images of 3368 × 2668 μm for each condition in three different tumors.

### Immunofluorescence

Tumor samples were frozen in OCT compound (Tissue-Teck) on liquid nitrogen. Eight μm thick sections were cut on a cryostat. Slides were fixed in ice cold acetone for 5 minutes, washed three times for five minutes in PBS and blocked in 10% donkey serum for 10 minutes. Incubation with anti-CD31 (1:50) and anti-VEGFR-2 (1:50) antibodies diluted in PBS/0.1% BSA was performed for 60 minutes. Subsequently, slides were washed three times for five minutes with PBS and incubated with donkey anti-rat 488 (1:500) and donkey anti-rabbit (1:500) secondary antibodies diluted in PBS/0.1% BSA for 30 minutes. Following three washes with PBS, slides were incubated for 10 minutes with DAPI solution (1:3000) and coverslipped using DAKO fluorescence mounting medium (#S3023). Slides were visualized using an inverted fluorescence microscope (Axiovert, Zeiss) and photographs were taken using a Zeiss AxioCam camera. Three random fields at 200 *×* magnification per xenografts (*n* = 3/group) were taken and the percentage of CD31/VEGFR-2 positive vessels was determined.

### Mouse model

Animal experiments were in accordance with the Swiss federal animal regulations and approved by the local veterinary office. Female nude or female C57BL/6 eight-week old mice were purchased from Janvier Labs (Saint Berthevin Cedex, France). Mice were randomized into different groups (*n* = 5/group; groups “vehicle” - “bicarbonate” - “sunitinib” - “bicarbonate and sunitinib”). HT29 (3 × 10^6^) or MC-38 (1 × 10^6^) cells were injected subcutaneously into the right flank. Sodium bicarbonate was added to the drinking water at a concentration of 200 mmol/L, starting one day before cancer cell injection. Once the tumor xenografts reached a mean volume of 25 mm^3^, mice were treated once daily with sunitinib (sunitinib 40 mg/kg p.o. diluted in 100 μl of carboxymethylcellulose 0.5%, NaCl 1.8%, Tween20 0.4% and ethahol 0.9% in distilled water, pH adjusted to 6.0) or vehicle (100 μl of carboxymethylcellulose 0.5%, NaCl 1.8%, Tween20 0.4% and ethanol 0.9% in distilled water, pH adjusted to 6.0). Tumor volumes were measured daily using a caliper and calculated with the formula V = A * B * C * π/6 where A is the length, B the width and C the height of the tumor. Animals were sacrificed once the biggest tumor of vehicle treated mice reached the size of 1’000 mm^3^ (defined as interruption criterion according to veterinary recommendations).

### Statistics

Statistical analysis including Student’s *t-test*, One-way ANOVA and Two-way ANOVA were carried out as appropriate using GraphPad Prism version 6.05.

## SUPPLEMENTARY MATERIALS FIGURES


