# Supplementary material for: Acidic pH reduces VEGF-mediated endothelial cell responses by downregulation of VEGFR-2; relevance for anti-angiogenic therapies
Source: Oncotarget. 2016 Nov 12;7(52):86026–38. doi: 10.18632/oncotarget.13323 (PMC5349894; doi:10.18632/oncotarget.13323)
Supplement: Supplementary file 1 [file oncotarget-07-86026-s001.pdf]

Acidic pH reduces VEGF-mediated endothelial cell responses by downregulation of VEGFR-2; relevance for anti-angiogenic therapies

Supplementary Materials

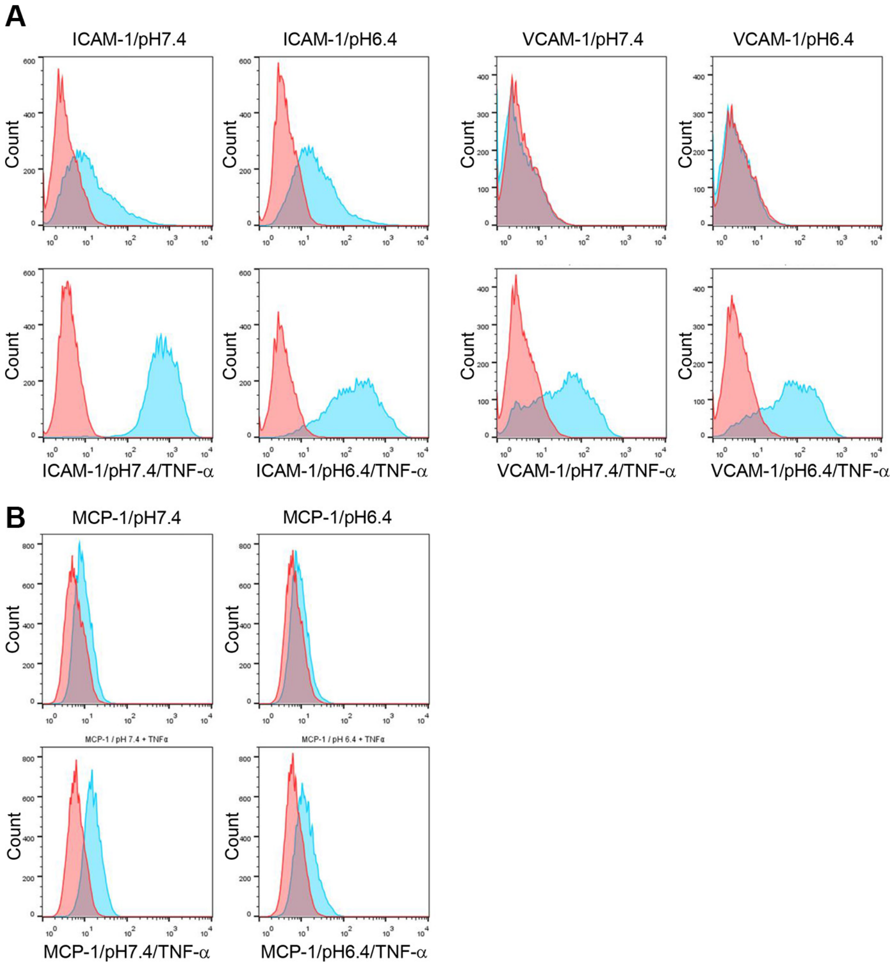

Supplementary Figure S1: Acidity does not inhibit TNF-α-mediated EC responses. (**A**) EC were cultured for 24 hours at pH 7.4 or 6.4 and subsequently stimulated or not with TNF-α (10 ng/ml) for 12 hours. EC were collected and stained with VCAM-1 or ICAM-1 antibody and analyzed by flow cytometry. Histograms show the relative fluorescence intensity of cells. The red histogram represents the isotype control antibody, the blue histogram represents VCAM-1 (right panels) or ICAM-1 antibody (left panels). (**B**) EC were cultured for 24 hours at pH 7.4 or 6.4 and subsequently stimulated or not with TNF-α (10 ng/ml) for 12 hours. EC were collected, fixed and permeabilized and intra-cellular MCP-1 was assessed by flow cytometry. Histograms show the relative fluorescence intensity of cells. The red histogram represents the isotype control antibody, the blue histogram represents MCP-1 antibody.

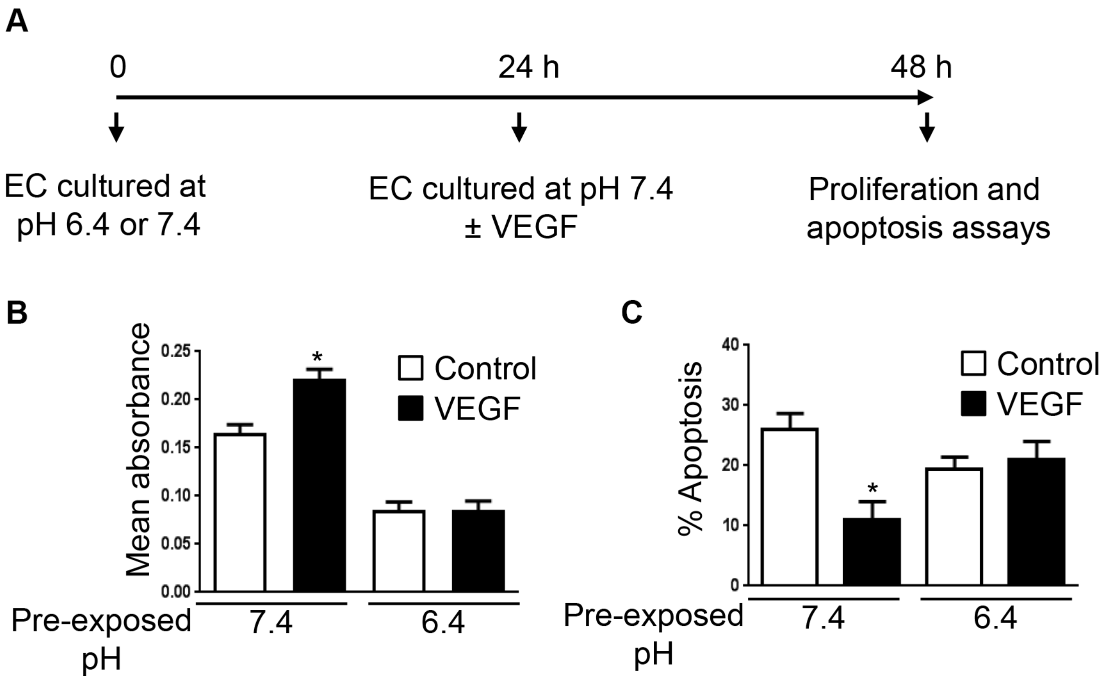

Supplementary Figure S2: Pre-exposition of EC to acidity inhibits VEGF-mediated EC responses. (**A**) Experimental design. EC were cultured for 24 hours at pH 7.4 or 6.4. EC were collected, plated and cultured in complete medium at pH 7.4 containing or not VEGF (10 ng/ml). MTS proliferation assay and apoptosis assay were performed after 24 hours stimulation with VEGF. For apoptosis assay, serum free medium pH 7.4 ± VEGF was used instead of complete medium. (**B**) MTS proliferation assay as described in panel A. Results are expressed as mean absorbance at 490 nm of triplicates ± 1 SD. *$p < 0.05$, Student's *t*-test, compared to control EC cultured at pH 7.4. (**C**) Percentage of EC undergoing apoptosis following the withdrawal of serum and processed as described in panel A. Results are expressed as mean apoptosis percentage ± 1 SD of three independent experiments. *$p < 0.05$, Student's *t*-test, compared to control EC cultured at pH 7.4.

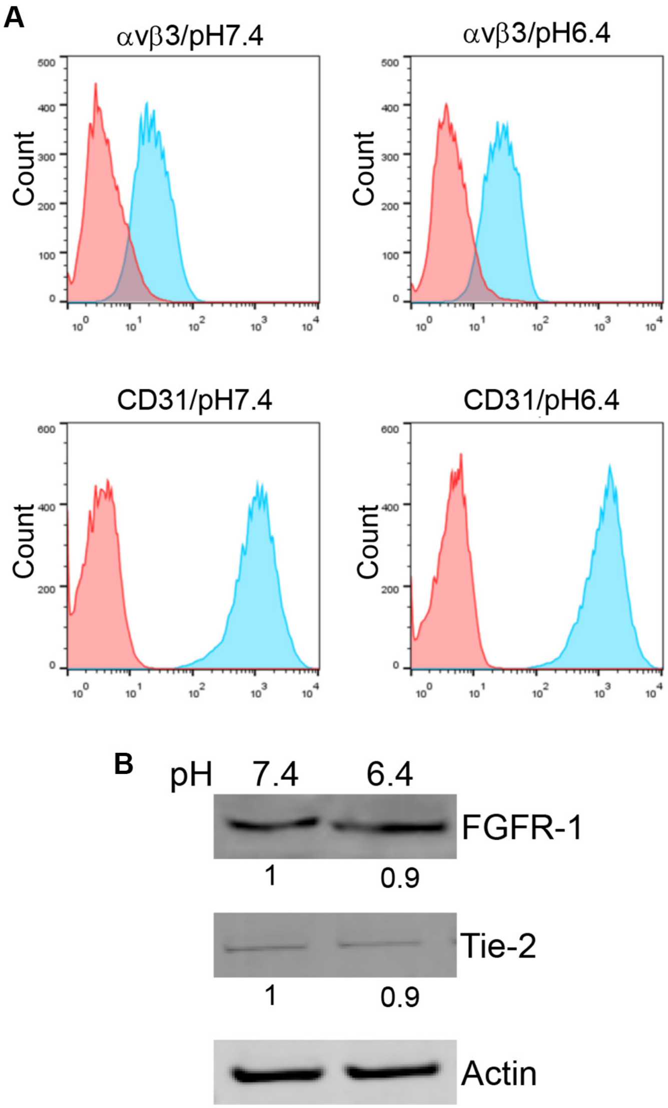

Supplementary Figure S3: Acidity does not reduce expression levels of αvβ3 integrin, CD31, FGFR-1 or Tie-2. (**A**) EC were cultured for 24 hours at pH 7.4 or 6.4. EC were collected and stained with αvβ3 integrin and CD31 antibodies and analyzed by flow cytometry. Histograms show the relative fluorescence intensity of cells. The red histogram represents the isotype control antibody, the blue histogram represents αvβ3 integrin (upper panels) or CD31 (lower panels) antibody. (**B**) EC were cultured for 24 hours at pH 7.4 or 6.4. Cell lysates were analyzed by Western blot for FGFR-1, Tie-2 and actin expression. Densitometric values of the ratio of FGFR-1 to actin or Tie-2 to actin are listed below the blots.

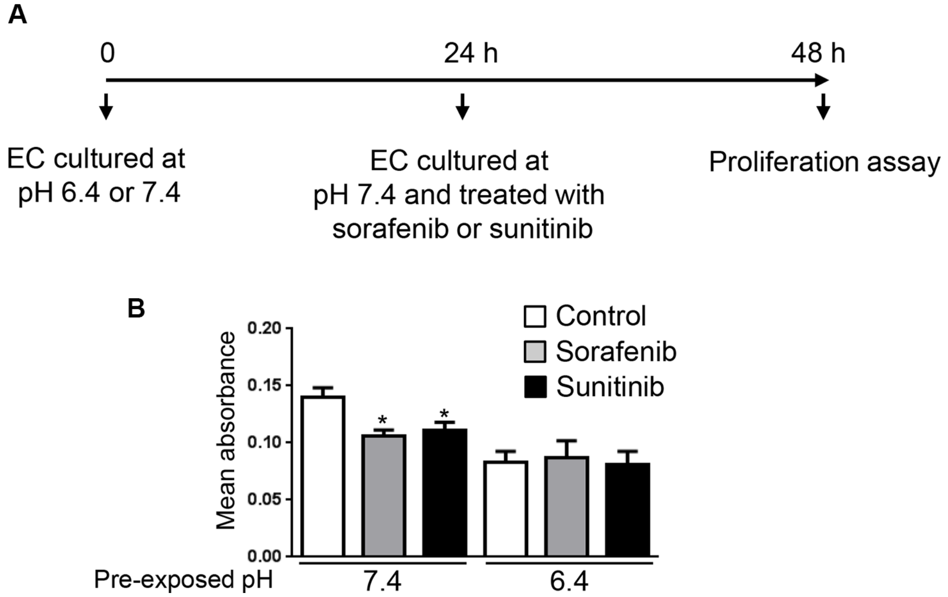

Supplementary Figure S4: Pre-exposition of EC to acidity decreases the anti-proliferative effects of sorafenib and sunitinib. (**A**) Experimental design. EC were cultured for 24 hours at pH 7.4 or 6.4. EC were collected and plated in complete medium at pH 7.4 containing or not sorafenib (1 µM) or sunitinib (1 µM). MTS proliferation was performed after 24 hours of treatment. (**B**) MTS proliferation assay as described in panel a. Results are expressed as mean absorbance at 490 nm of triplicates ± 1 SD. **p < 0.05*, Student's *t*-test, compared to untreated EC cultured at pH 7.4.
